# Opposite environmental gating of the experienced utility (‘liking’) and decision utility (‘wanting’) of heroin versus cocaine in animals and humans: implications for computational neuroscience

**DOI:** 10.1007/s00213-019-05318-9

**Published:** 2019-07-09

**Authors:** Aldo Badiani, Daniele Caprioli, Silvana De Pirro

**Affiliations:** 1 Department of Physiology and Pharmacology, Sapienza University of Rome, Rome, Italy; 2 Sussex Addiction Research & Intervention Centre (SARIC) and School of Psychology, University of Sussex, Brighton, UK; 3 Santa Lucia Foundation (IRCCS Fondazione Santa Lucia), Rome, Italy

**Keywords:** Heroin, Cocaine, Reward, Pleasure, Motivation, Utility, Opioid, Psychostimulant, Addiction

## Abstract

**Background:**

In this paper, we reviewed translational studies concerned with environmental influences on the rewarding effects of heroin versus cocaine in rats and humans with substance use disorder. These studies show that both experienced utility (‘liking’) and decision utility (‘wanting’) of heroin and cocaine shift in opposite directions as a function of the setting in which these drugs were used. Briefly, rats and humans prefer using heroin at home but cocaine outside the home. These findings appear to challenge prevailing theories of drug reward, which focus on the notion of shared substrate of action for drug of abuse, and in particular on their shared ability to facilitate dopaminergic transmission.

**Aims:**

Thus, in the second part of the paper, we verified whether our findings could be accounted for by available computational models of reward. To account for our findings, a model must include a component that could mediate *the substance-specific influence of setting on drug reward*

**Results:**

It appears of the extant models that none is fully compatible with the results of our studies.

**Conclusions:**

We hope that this paper will serve as stimulus to design computational models more attuned to the complex mechanisms responsible for the rewarding effects of drugs in real-world contexts.

## Introduction

It is well known that, under certain conditions, some individuals eagerly self-administer drugs such as heroin and cocaine. The rewarding effects of these drugs differ from those of natural stimuli because drugs can act on the relevant circuitry of the brain without first stimulating one of the five major senses and in the absence of specialized homeostatic mechanisms regulating their intake. In the past five decades, a vast body of work has clarified in great detail the molecular targets of most abused substances, but the exact mechanisms responsible for their rewarding effects are still not clear from both a neurobiological and conceptual point of view. The nature of some of the obstacles that make this aim difficult to achieve will become clear in the remaining of this paper. For the moment, we list three issues that might sound surprising to some readers. First, the neurobiological mechanisms responsible for the euphoriant effects of drugs (‘drug pleasure’) are poorly understood. For example, there is considerable evidence indicating that the mesolimbic dopaminergic system does not mediate pleasure or ‘drug pleasure’ (for reviews, see Robinson and Berridge 1993; Berridge 2012; Berridge and O’Doherty 2014), but it is less appreciated that it even may not play a *necessary* role in the reinforcing effects of drug such as opioids and alcohol (see Badiani et al. 2011; Nutt et al. 2015), or even in drug reward in general (see Ikemoto 2010). Second, the behavioral and neurobiological effects of drugs are powerfully modulated by the circumstances surrounding drug use (e.g., Zinberg 1984; Stewart et al. 1984; Stewart and Badiani 1993; Badiani et al. 1995a, 1998, 1999; Kendler et al. 2003; Paolone et al. 2004; Badiani and Robinson 2004; Robinson and Kolb 2004; Badiani 2013). Third, despite the explosion of research in artificial intelligence and computational modeling, our understanding of the neural algorithms regulating motivated behavior in the real world is still extremely rudimentary. This is particularly true of drug-taking behavior, as relatively little work has focused on the rewarding effects of drugs from a computational point of view.

In this paper, we will first review translational studies showing that the setting of drug use can influence in opposite directions the rewarding effects of opioids (e.g., heroin) versus psychostimulants (e.g., cocaine) in rats and humans. The findings from these studies challenge the still prevailing unitary notion of drug reward that focus on the shared ability of drugs to activate dopaminergic transmission (e.g., Wise 1980; Di Chiara and Imperato 1988; Robinson and Berridge 1993; Nestler 2001, 2004; Hyman et al. 2006; Koob and Volkow 2010; Berridge 2012; Covey et al. 2014; Keiflin and Janak 2015; Volkow and Morales 1995; Volkow et al. 2017; Berridge and Robinson 2016; Keramati et al. 2017). This notion is so entrenched that, despite robust evidence to the contrary (see Badiani et al. 2011, 2018; Nutt et al. 2015), it is portrayed as a fact even in policy-making documents (Surgeon General’s Report on the consequences of alcohol and drug abuse on health 2016; https://addiction.surgeongeneral.gov/). The aim of this paper is therefore to verify whether our findings could be accounted for by available computational models of drug reward.

## Defining ‘drug reward’: experienced utility versus decision utility

The reported reasons for using drugs change from drug to drug, from individual to individual, and from situation to situation (e.g., see Gossop and Connell 1975; Harford 1978). Unsurprisingly, the drug effect that has attracted most research interest is represented by the intense pleasure produced by the drug such as heroin or cocaine (at least in some individuals). However, drugs produce a myriad of other effects that can lead an individual to use one or more of them (see, for example, Müller and Schumann 2011). Although only in a minority of cases the pattern of use becomes compulsive, leading to a diagnosis of substance use disorder (SUD), drugs such as heroin, cocaine, and alcohol are commonly referred to as addictive drugs. Unfortunately, this tends to produce a confusion between the mechanisms responsible for the rewarding effects of drugs and the mechanisms responsible for the loss of control over their use, that is, drug addiction. In the present paper, we are concerned with the former rather than with the latter.

Another source of confusion is represented by the term ‘reward’. This term is used by some to indicate the “recompense given after a particular response which reinforces learning or behaviour” (OED 2019). According to this meaning, ‘reward’ is synonymous with ‘reinforcer,’ which is a purely operational term that does not require making assumptions about underlying psychological processes. Others use the term reward to indicate the positive hedonic effect of rewarding stimuli (e.g., Redish et al. 2008). Still others indicate with the term ‘reward’ the entire set of hypothetical psychological construct(s) responsible for the rewarding effects (e.g., Berridge et al. 2003; Berridge 2012).^1^ In the present paper, we use reward according to the last meaning, which coincides with the notion of utility (Kahneman et al. 1997; Kahneman 1999).

Furthermore, we adopted here the distinction, proposed independently by Berridge and colleagues (Robinson and Berridge 1993; Berridge et al. 2009) and by Kahneman and colleagues (Kahneman et al. 1997; Kahneman 1999), between two main components of reward.^2^ Berridge and colleagues, building on Bindra’s model of motivation (Bindra 1976), distinguished *liking* (pleasure/displeasure) from *wanting* (incentive salience). Kahneman and colleagues, building on Bentham’s economic theory of utility (Bentham 1823), distinguished *experienced utility* (a psychological construct indicating the hedonic experience) and *decision utility* (a computational quantity inferred from observed choices, such as purchasing a good). The concepts of experienced utility and decision utility appear to be germane to those of *liking* and *wanting*, as acknowledged by Berridge and colleagues (Zhang et al. 2009; Berridge and O’Doherty 2014). However, Berridge’s *liking* and *wanting* indicate core motivational processes, whereas Kahneman’s experienced and decision utility are much broader concepts, including both emotional and cognitive processing (see Berridge and O’Doherty 2014, pp. 341–342).

Both Berridge’s and Kahneman’s theoretical frameworks center on the notion that the two components of reward/utility are mediated by independent processes, and both stipulate that it is conceptually wrong to automatically infer one from the other, as they are dissociable. Some researchers have dismissed the concepts of experienced utility based on the assumption that subjective hedonic experience cannot be observed or measured and that “choices provide all necessary information about the utility of outcomes because rational agents who wish to do so will optimize their hedonic experience” (Kahneman et al. 1997, p. 375). At present, this simplification is endorsed by a minority of scholars and the basic distinction between experienced utility and decision utility has been adopted by most researchers in the field. Yet, it is still often the case that, even in scholarly papers, affect is more or less surreptitiously inferred from action or vice versa (for a discussion of this issue, see Berridge 2003, p. 115). As discussed below, experienced utility can be estimated via “objective responses (including affective behavioral reactions, physiological autonomic, and brain limbic reactions) as well as in humans at least, subjective feelings reported as pleasure” (Berridge and O’Doherty 2014, p. 336).

The following sections summarize the findings of experiments aimed at measuring the experienced utility and the decision utility of heroin versus cocaine in different settings in rats and humans. By necessity, the experimental procedures used to assess experienced drug utility in rats and humans were complementary rather than identical. Whenever possible, a within-subject design was used, to ensure that the differences observed in the utility of heroin versus cocaine were not due to individual variability in the response to the two drugs.

## Experienced utility of heroin versus cocaine in rats and humans

The simplest form of experienced utility is represented by *instant utility* (“a measure of hedonic and affective experience, which can be derived from immediate reports of current subjective experience or from physiological indices”, Kahneman et al. 1997, p. 376). However, in most cases, the hedonic experience has a temporal dimension and might change quantitatively and/or qualitatively over time, which makes it appropriate to consider the experienced utility of *temporally extended outcomes* (TEOs). The term TEO is self-explanatory, indicating the overall utility of a series of stimuli distributed over time that are perceived as part of the same outcome, so that “when an evaluative summary of a temporally extended outcome is required, a representative moment that stands for the entire outcome is selected or constructed; the temporally extended outcome is then assigned the value of its representative moment” (Kahneman 2000)*.* Indeed, the experienced utility of TEOs can be measured as *total utility*, calculated from the temporal profile of instant utility or, more frequently, as *remembered utility*, which encompasses the overall hedonic experience,^3^ and determines “whether a situation experienced in the past should now be approached or avoided” (Kahneman et al. 1997, p. 380).

### Instant utility of heroin versus cocaine in rats

Rats emit ultrasonic vocalizations (USVs) in the range of 50 kHz in response to rewarding stimuli, such as intra- and heterospecific play (Knutson et al. 1998; Burgdorf and Panksepp 2001; Mällo et al. 2007; Panksepp and Burgdorf 2000, 2003; Schwarting et al. 2007; Wöhr et al. 2009), sex (McGinnis and Vakulenko 2003; White et al. 1990; Bialy et al. 2000), food (Burgdorf et al. 2000), electrical stimulation of the medial forebrain bundle (Burgdorf et al. 2000), and addictive drugs (Ahrens et al. 2009; Knutson et al. 1999; Natusch and Schwarting 2010; Wintink and Brudzynski 2001; Wright et al. 2010; Barker et al. 2010; Maier et al. 2010). Based on this evidence, it has been proposed that 50-kHz USVs reflect positive affective states in the rat (Knutson et al. 2002).^4^ Therefore, in the study described below (Avvisati et al. 2016), we quantified the instant utility of heroin versus cocaine in rats by measuring the emission of 50-kHz USVs after intravenous self-administration of either drug.

We first trained two groups of male Sprague–Dawley rats to self-administer heroin and cocaine (on alternate days for 2 weeks) either at home or outside the home. The rats tested at home were individually housed in standard two-lever self-administration chambers, where they remained for the entire duration of the experiment. The other rats were individually housed in standard transparent plastic cages and were transferred to the self-administration chambers immediately before the start of each testing session. Notice that throughout the experiments, the rats were individually housed and tested in the same dedicated testing room (thus, no transport from one room to another and no disruption of social context or circadian rhythmicity) with ad libitum access to food and water (except during the test sessions). Thus, although the self-administration environment was physically identical for the two groups, one group experienced the drugs at home and the other group outside the home.

**Fig. 1:**
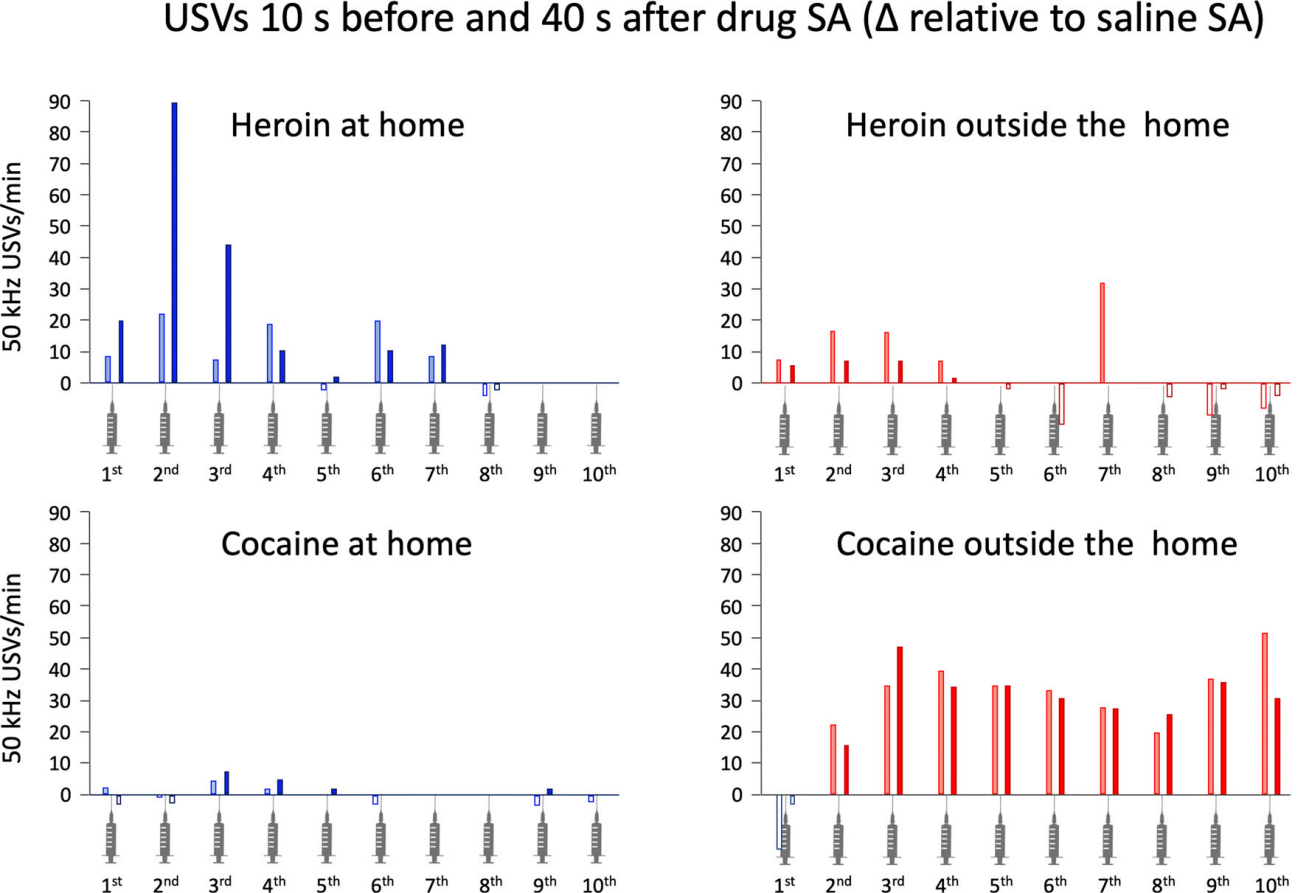
In this within subject experiment, rats were trained to self-administer heroin (25 μg/kg per infusion) and cocaine (400 μg/kg per infusion), on alternate sessions, either at home or outside the home (see text for details). The training lasted for 14 sessions. On sessions 15 and 16, the rats self-administered saline. Ultrasonic vocalizations (USVs) in the range of 50 kHz were recorded on sessions 13–16. The graph shows the mean net number of 50-kHz USVs emitted 10 s before and 40 s after each of the first ten infusions of heroin and cocaine. This was done by subtracting the number of USVs emitted before and saline self-administration from the number of USVs emitted before and after the corresponding drug infusion. For example, the number of vocalizations produced before and after the first infusion of saline when the rats pressed on the heroin-paired lever (on sessions 15 or 16) was subtracted from the corresponding values for the first infusion of heroin (on sessions 13 or 14), and so no (for more details see Avvisati et al. 2016, Figures 7 and 8).Rats tested at home emitted more 50-kHz USVs before and after heroin than before and after cocaine. The opposite was observed in rats tested outside the home. Data from Avvisati et al. (2016)

On the last two sessions of drug-self-administration (sessions 13 and 14), USVs were recorded. The same was done on two subsequent sessions (sessions 15 and 16) during which the rats self-administered saline solution. The number of vocalizations emitted during sessions 15 and 16 provided a baseline measure incorporating both spontaneous vocalization and conditioned vocalization in response to heroin- versus cocaine-paired cues (i.e., cue lights, pump noise, vehicle infusion). We reasoned that a net increase in 50-kHz USVs after each infusion would be largely dependent on a positive affective state produced by the drug. Indeed, we found that the instant utility of heroin and cocaine varied as a function of the context of self-administration but in *opposite* directions. Rats that had self-administered the drugs in their home environment emitted more vocalizations after heroin than after cocaine (see Fig. 1). The opposite was observed in rats that had self-administered the same drugs in a distinct nonhome environment (that is, outside the home). These rats emitted more vocalization after cocaine than after heroin. In summary, these data appear to suggest that the instant utility of heroin is greatest at home, whereas the instant utility of cocaine is greatest outside the home.

### Remembered utility of heroin versus cocaine in humans

We recently completed a study (De Pirro et al. 2018), in which we quantified the experienced utility of cocaine versus heroin use in female and male individuals with substance use disorder (SUD). The participants were recruited among the clients of a public substance misuse service affiliated to the International Red Cross Red Crescent (Villa Maraini Foundation, Rome, Italy), and all of them had a long history (about 15 years on average) of both heroin and cocaine misuse and no major psychiatric comorbidity.

As we could not record instant utility in real-world settings, we quantified instead the remembered utility of the TEO of drug use. As pointed out by Kahneman and colleagues (Kahneman et al. 1997; Kahneman 1999) and by Berridge and colleagues (Berridge and O’Doherty 2014; Berridge and Kringelbach 2013), retrospective reports of remembered utility might not provide a veridical recall of actual past pleasures, as they might represent active mnemonic reconstructions susceptible of distortion. More reliably, remembered utility can be measured by quantifying “affective behavioral reactions, physiological reactions, and brain limbic reactions” (Berridge and O’Doherty 2014, p. 336), during the recollection of past drug experiences.

**Fig. 2:**
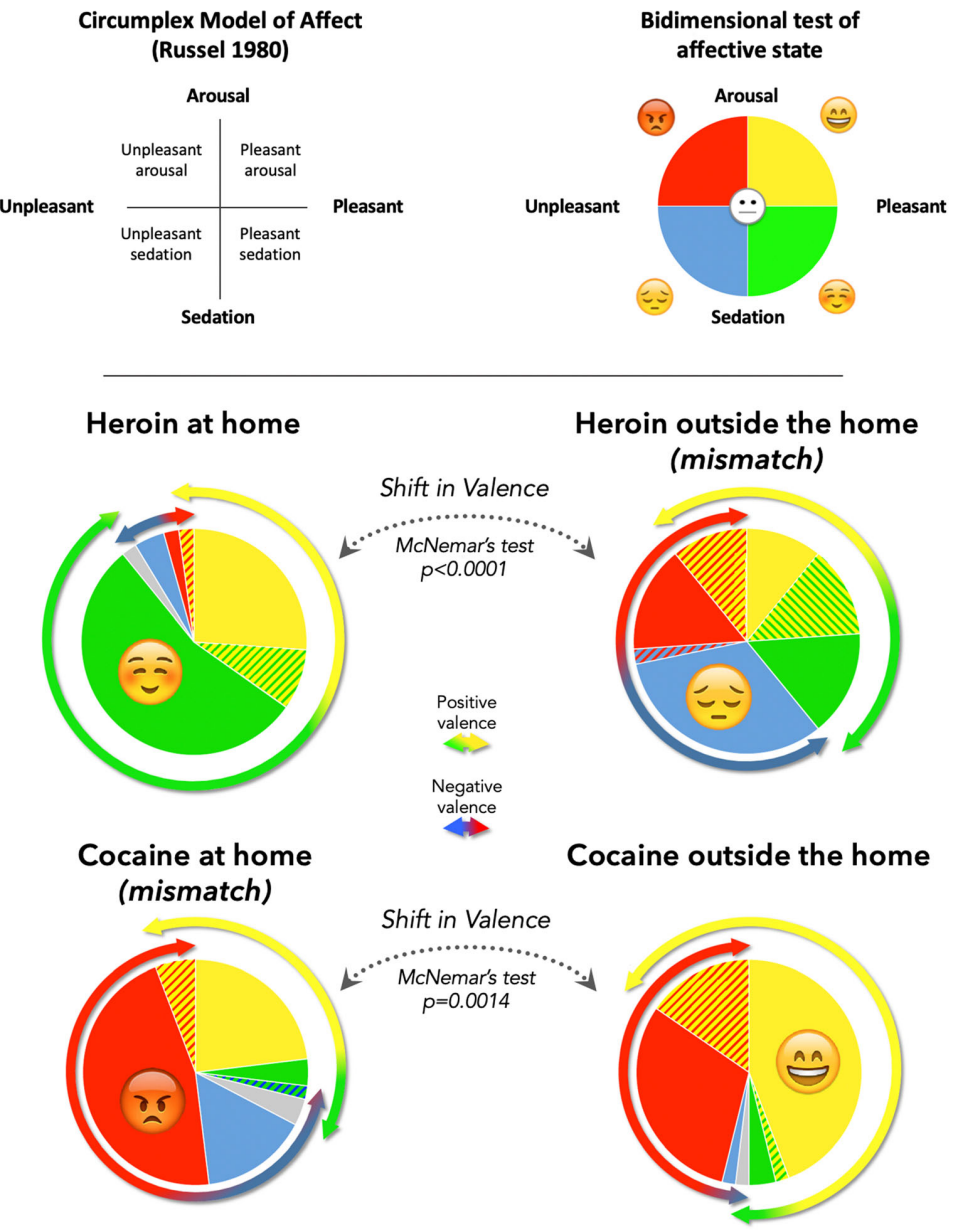
In this within-subject experiment, we assessed the affective state induced by heroin and cocaine as reported by individuals with addiction to both drugs. Top-left, graphic representation of the circumplex model of affect (Russell 1980). Top-right, bidimensional representation of affective states used developed based on the circumplex model of affect (on the left) by removing the labels indicating different levels for each dimension and by adding emoticons. The middle and bottom pie charts indicate the subjective appraisal of the emotional valence of drug experience as a function of drug and setting. Each pie chart represents the proportion of participants reporting the affective states after heroin or cocaine use, either at home or outside the home (see text for details). The McNemar’s test indicated significant shifts in valence as a function of the setting of drug use. A small proportion of participants reported two affective states (hatched lines) or more (gray). Data redrawn from De Pirro et al. (2018)

Thus, in our study, we used an emotional task based on the circumplex model of affect (Russell 1980), which posits that all affective states arise from the interaction of two core neurobiological processes: arousal (along high–low energy continuum) and valence (along a pleasure–displeasure continuum). Accordingly, the graphic task represented a two-dimensional space with arousal on the vertical dimension and valence on the horizontal dimension (Fig. 2, top panels). Emoticons and colors were added to increase the evocative power of the diagram (Nathanson et al. 2016; Kaye et al. 2017). The participants were asked to select the quadrant of the diagram that best corresponded to the affective state experienced under the influence of heroin or cocaine. By virtue of being fast, user-friendly, and intuitive (and dispensing altogether with verbal descriptions), the task minimized the risk of cognitive distortions in the recollection of affective states (e.g., Kahneman et al. 1997; Kahneman 1999; Robinson and Clore 2002; Gorlin et al. 2019), and in particular, the framing effects deriving from the participants’ negative feelings toward their own addiction (Dearing et al. 2005; Luoma et al. 2012, 2013).

The experienced utility of heroin and cocaine in humans varied as a function of context and also in this case in *opposite* directions. The remembered utility of heroin was in fact much greater at home than outside the home, whereas the remembered utility of cocaine was greater outside the home than at home (see Fig. 2, middle and bottom panels). To the extent that the remembered utility of heroin and cocaine reflects the total utility of TEO, this pattern appears to be the same as that seen in rats.

Additional evidence that the remembered utility of heroin and cocaine is influenced in opposite directions by the setting of use is provided by a functional magnetic resonance imaging (fMRI) study in which individuals with SUD were asked to re-evoke the hedonic experience of using heroin versus cocaine at home or outside the home (De Pirro et al. 2018). In addition to the expected changes in blood-oxygenation-level-dependent (BOLD) signal in regions that have been previously implicated in the retrieval of memories (angular gyrus; Bonnici et al. 2016; Kuhl and Chun 2014; Richter et al. 2016) and in mental imagery (precuneus; Fletcher et al. 1995; Richter et al. 2016), we found a *double dissociation, as a function of substance and setting,* in the pattern of activation of regions implicated in brain reward (PFCx and the striatum; Goldstein and Volkow 2002, 2011; Cox et al. 2009; Volkow et al. 2017; Leyton and Vezina 2013) and the in contextualization of emotional processing (cerebellum; Schmahmann 1996, 2004; Schmahmann and Sherman 1998; Scheuerecker et al. 2007; Stoodley 2012; Adamaszek et al. 2014, 2017; Van Overwalle et al. 2015), that is in the fronto-striato-cerebellar circuit (Hoshi et al. 2005; Bostan et al. 2010; Bostan and Strick 2018).

Another interesting finding of the study by De Pirro et al. (2018) is that the remembered utility of neither heroin nor cocaine was ever entirely positive. Actually, in certain settings, cocaine produced a mainly unpleasant affective state in almost two thirds of users, consistent with previous reports of cocaine-induced aversive states in both rodents and humans (Geist and Ettenberg 1997; Ettenberg et al. 1999; Knackstedt et al. 2002; Anthony et al. 1989; Geracioti Jr and Post 1991; Breiter et al. 1997). Thus, the fact that a drug is willingly used (indicating decision utility) does not necessarily imply that its experienced utility is positive.

## Decision utility of heroin versus cocaine in rats and humans

Decision utility is “inferred from choices, either by direct comparisons of similar objects or by indirect methods, such as elicited willingness to pay” (Kahneman et al. 1997, pp. 376–377). In all our experiments, drug intake required ‘willingness to pay’: in terms of workload in rats (except for the noncontingent administration of drug ‘primings’), in terms of money, or some type of barter, in humans. In some experiments, there was a ‘direct comparison’ between ‘similar objects,’ that is, between heroin and cocaine. In all experiments, the decision utility of heroin and/or cocaine was assessed in one of two settings.

### Decision utility in rats

A series of experiments (Caprioli et al. 2007a, 2008, 2009; Celentano et al. 2009; Montanari et al. 2015; Avvisati et al. 2016; De Luca et al. 2019) were conducted in male Sprague–Dawley rats to assess the decision utility of heroin versus cocaine (or amphetamine). Also in these experiments, the rats were tested either at home or outside the home. Decision utility was assessed using different procedures.

**Fig. 3:**
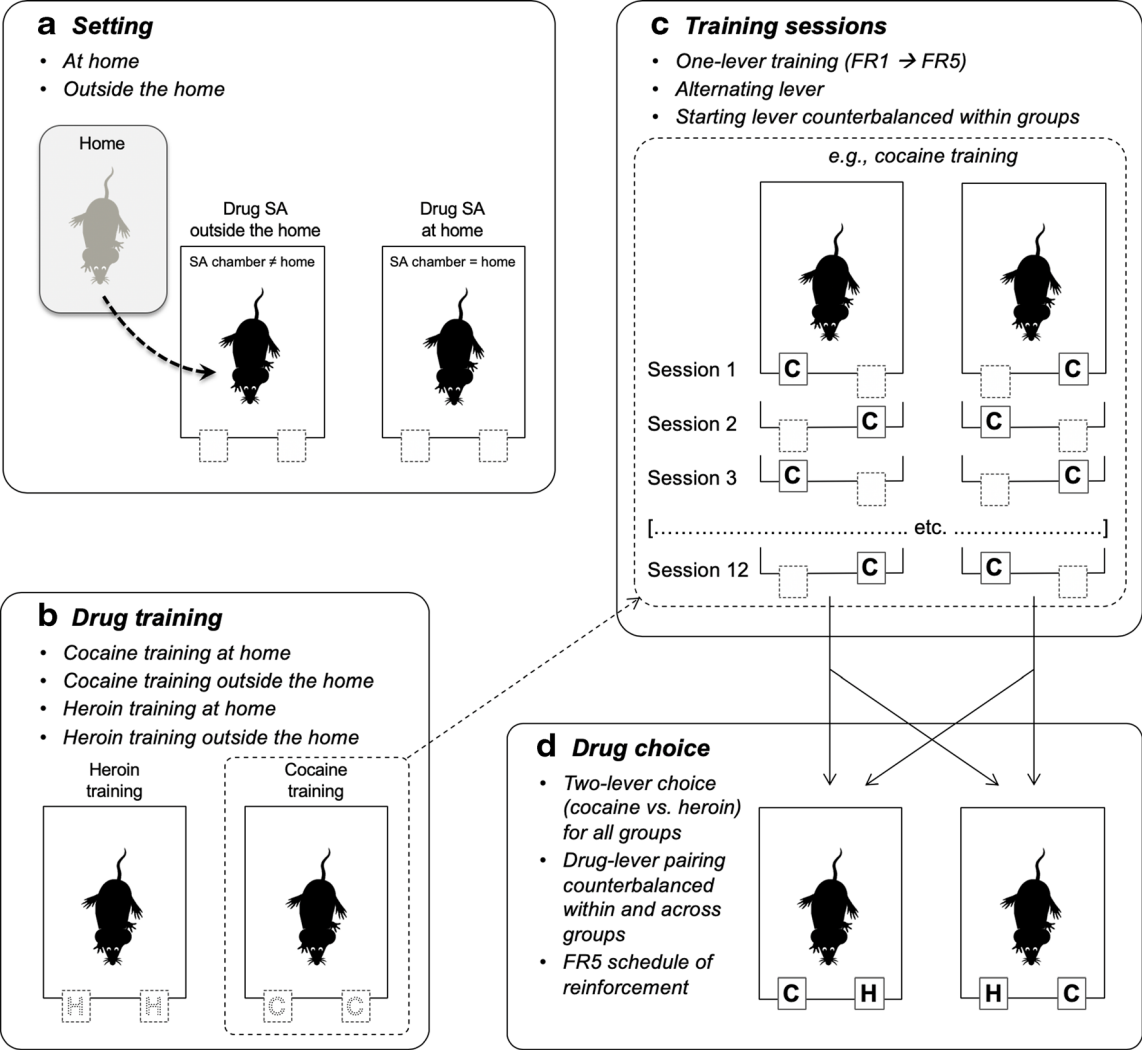
Experimental design of a within-subject study concerned with heroin versus cocaine choice in rats. A-C The rats were first trained to self-administer heroin (25 μg/kg per infusion) and cocaine (400 μg/kg per infusion), on alternate sessions, either at home or outside the home. The training lasted for 12 sessions (see Fig. 4 for results). D The rats were then given the opportunity to choose between heroin and cocaine for seven consecutive sessions. At the end of the choice sessions, the rats were classified as cocaine-preferring, heroin-preferring, or nonpreferring (see Fig. 5 for results). Modified from De Luca et al. (2019)

**Fig. 4:**
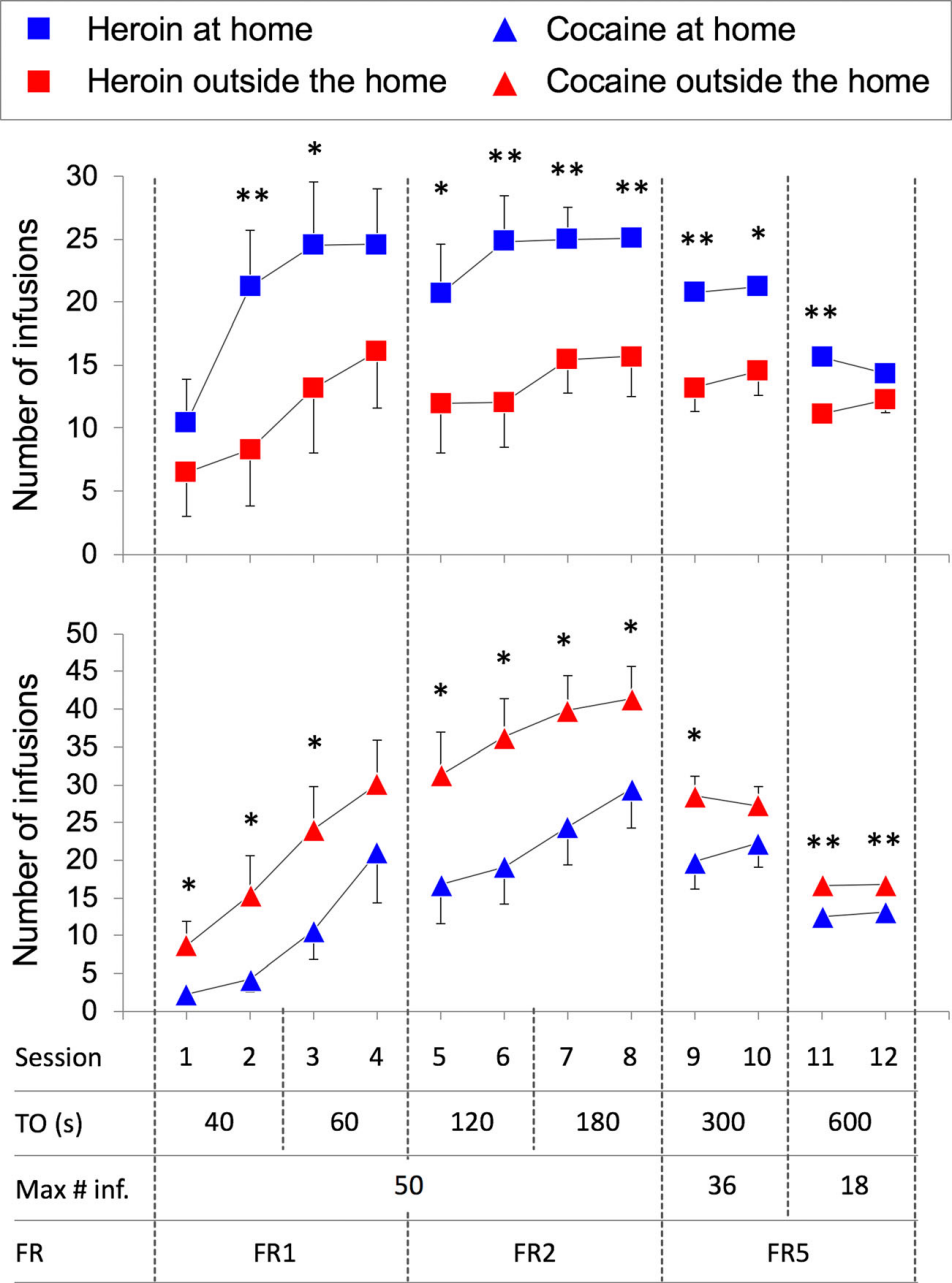
Rats were tested as described in Fig. 3A–C. Mean (±SEM) number of infusions during the training phase for the heroin- and cocaine-trained groups, as a function of setting, time-out (TO) period, maximum number of infusions, and fixed ratio (FR). Single and double asterisks indicate significant effect setting (*p* < 0.05 and *p* < 0.01, respectively). Consistent with previous findings (Caprioli et al. 2007a, 2008) and despite the constraints in the maximum number of infusions, rats at home self-administered more heroin than rats outside the home, whereas rats outside the home self-administered more cocaine than rats at home. Modified from De Luca et al. (2019)

**Fig. 5:**
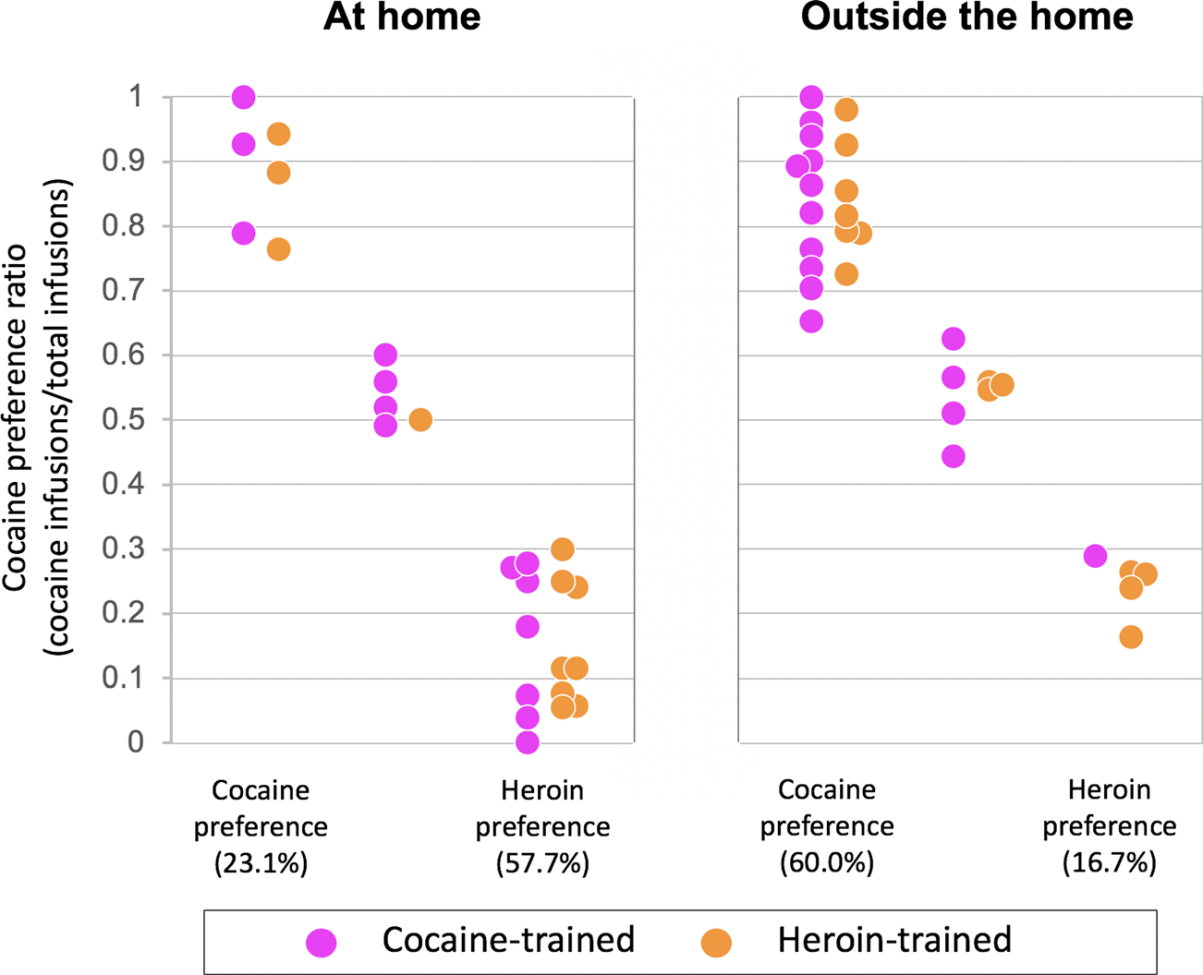
Rats were tested as described in Fig. 3D. Drug preferences in individual rats (calculated using bootstrapping analysis), as a function of setting and drug history (see text for details). The preference for one drug or the other was influenced in *opposite* directions by the context. At home, 57.7% rats preferred heroin to cocaine, whereas only 23.1% preferred cocaine to heroin. Outside the home, 60% rats preferred cocaine to heroin, whereas only 16.7% preferred heroin to cocaine. Some rats (19.2% at home and 23.3% outside the home) did not exhibit a significant preference for either drug. Modified from De Luca et al. (2019)

**Fig. 6:**
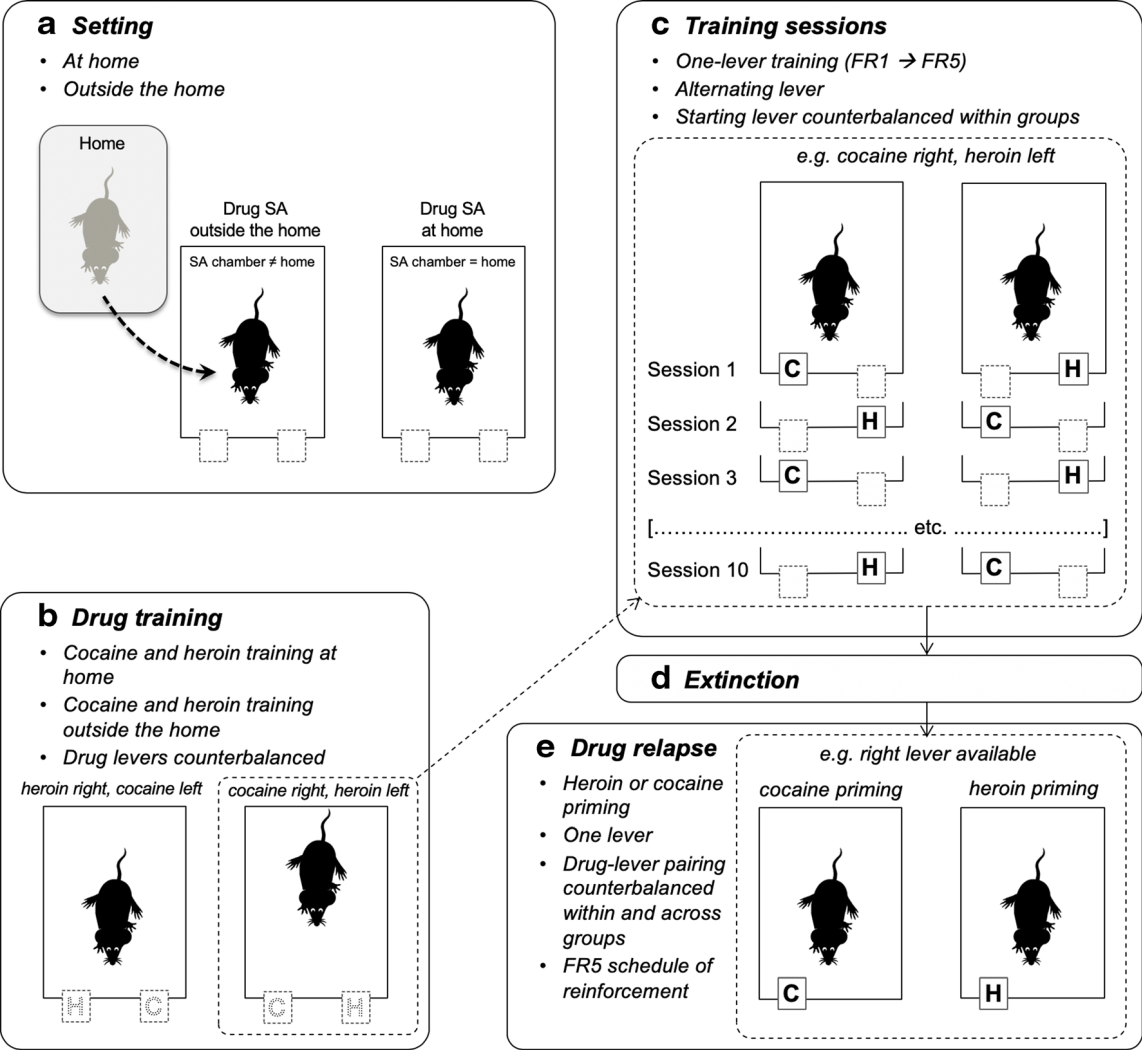
Experimental design aimed at quantifying the decision utility of heroin seeking and cocaine seeking after a period of abstinence from the drug (Montanari et al. 2015). The rats were first trained to self-administer heroin and cocaine on alternate days (A–C) and then underwent an extinction procedure (D), during which lever pressing did not result in drug infusion even in the presence of drug-paired cues (e.g., lever extension, cue lights, infusion of vehicle). The rats were then tested in a reinstatement procedure (E), developed to model relapse into drug seeking after a period of abstinence. The ability of a single, noncontingent intravenous drug infusion (drug priming) to precipitate drug seeking was assessed by comparing lever pressing during the reinstatement session to lever pressing under extinction conditions (for results, see Fig. 7)

**Fig. 7:**
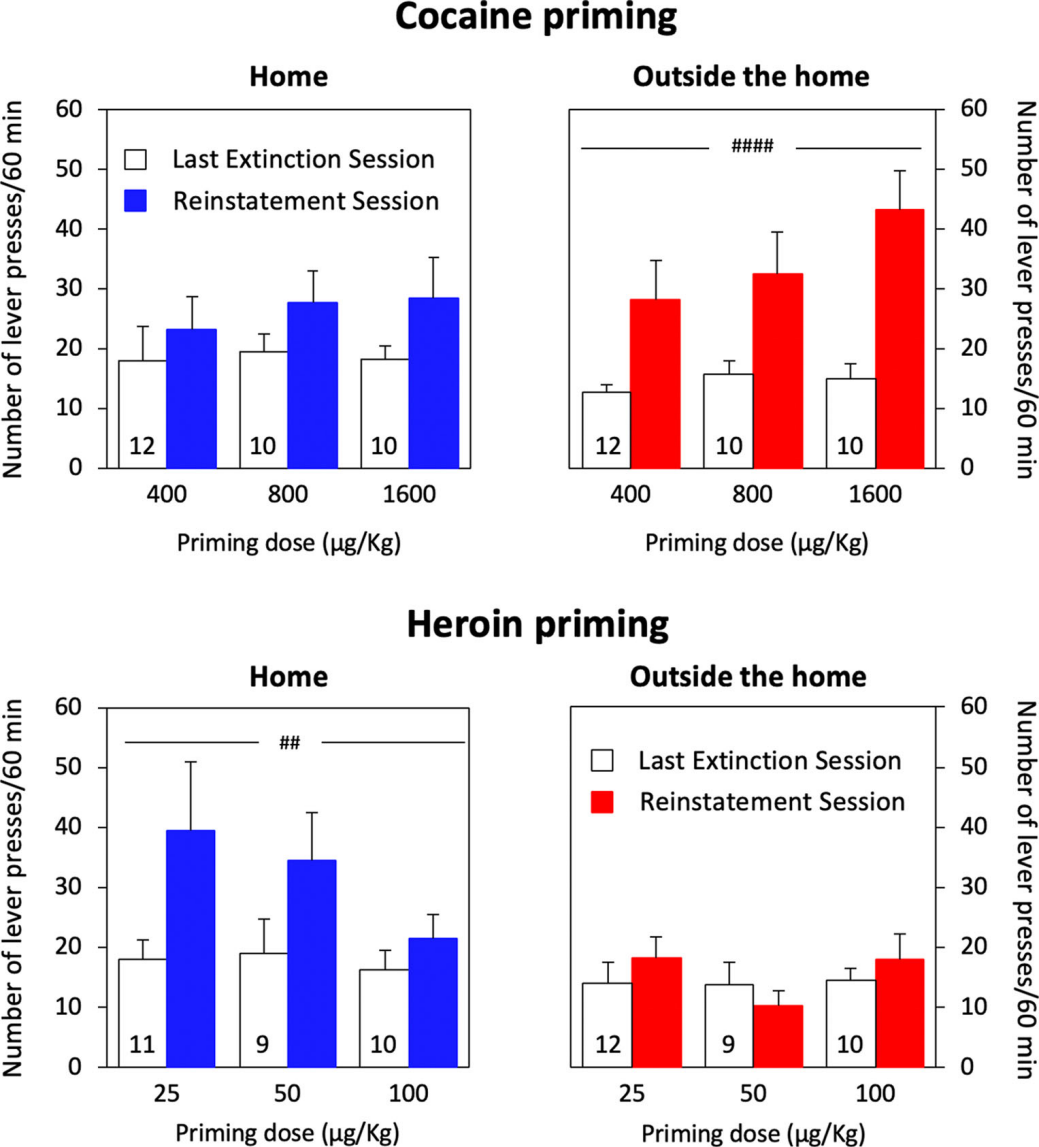
Mean (±SEM) number of lever presses during the first hour of the last extinction session (white bars) versus the reinstatement session (black bars) for rats tested at home versus rats outside the home (see Fig. 6). At the beginning of the reinstatement session, independent groups of rats (*N* values are indicated by the numbers within the white bars) received noncontingent intravenous (i.v.) infusions of one of three doses of cocaine (top panels) or heroin (bottom panels). Significant (##*p* ≤ 0.01 and ####*p* ≤ 0.0001) main effect of priming. Data from Montanari et al. (2015)

1. Between-subject procedures were used to assess the rats’ willingness to pay for heroin or cocaine, as a function of setting. In some experiments (Caprioli et al. 2007a, 2008), the rats were given the choice between a lever that triggered a drug infusion and a control lever that triggered an infusion of vehicle, and the work necessary to obtain the drug was increased progressively across sessions and within session, using a break-point procedure. The rats’ decision to self-administer heroin or cocaine was influenced in an *opposite* manner by the setting. Rats tested at home self-administered more heroin at home than rats tested outside the home. In contrast, the rats took more cocaine (and amphetamine) outside the home than at home. Furthermore, the rats worked harder for heroin at home than outside the home and for cocaine (or amphetamine) outside the home than at home.
2. Within-subject procedures were used to compare the rats’ willingness to pay for heroin versus cocaine, as a function of setting. In these experiments (Caprioli et al. 2009; Celentano et al. 2009; Montanari et al. 2015; Avvisati et al. 2016), the rats were trained to press on alternate days for heroin and cocaine. One lever was paired with heroin and the other with cocaine (in a counterbalanced fashion), and the work necessary to obtain each drug was increased progressively across sessions. Also, in this case, the rats’ decision was a function of context, which influenced in an *opposite* manner heroin versus cocaine intake, and of workload. The ratio of cocaine to heroin infusions was greater outside the home than at home (indirectly indicating a preference) and became progressively larger with the increase in workload.
3. Within-subject choice procedures were used in some studies to assess the rats’ preference for heroin or for cocaine. *To the best of our knowledge, these are the first and only studies to have directly compared the rewarding effects of heroin and cocaine.* In one of these studies (Caprioli et al. 2009), the rats were first trained to self-administer heroin and cocaine on alternate days, as previously described. The rats were then given the opportunity to choose between heroin and cocaine within the same session for several sessions. At the end of the choice sessions, the rats were classified, using a straightforward bootstrapping procedure (Wilson 1927; Newcombe 1988), as cocaine-preferring, heroin-preferring, or nonpreferring. The preference for one drug or the other was influenced in *opposite* directions by the context. At home, the rats tended to prefer heroin to cocaine; outside the home, the rats tended to prefer cocaine to heroin. Strikingly, the same double dissociation in decision-making was observed when we used an experimental design (see Fig. 3) in which rats were trained to receive the same drug (heroin for some, cocaine for other rats, Figs. 3A–C, 4) when pressing on either lever (De Luca et al. 2019). The rats were then offered the choice between heroin and cocaine, as described above (Fig. 3D). Also in this case, the rats tested at home tended to prefer heroin to cocaine, whereas the rats tested outside the home tended to prefer cocaine to heroin (Fig. 5). *In summary, drug preference appears to be influenced to a much greater extent by the context of drug use than by the history of drug use.*
4. We also quantified the decision utility of heroin seeking and cocaine seeking after a period of abstinence from the drug (Montanari et al. 2015). The rats were first trained to self-administer heroin and cocaine on alternate days (Fig. 6A–C) and then underwent an extinction procedure (Fig. 6D), during which lever pressing did not result in drug infusion even in the presence of drug-paired cues (e.g., lever extension, cue lights, infusion of vehicle). The rats were then tested in a reinstatement procedure (Fig. 6E), developed to model relapse into drug seeking after a period of abstinence (de Wit and Stewart 1981; Shaham et al. 2003). The ability of a single, noncontingent intravenous drug infusion (drug priming) to precipitate drug seeking was assessed by comparing lever pressing during the reinstatement session to lever pressing under extinction conditions. Heroin priming precipitated heroin seeking in rats tested at home but not in rats tested outside the home, whereas the *opposite* was observed for cocaine: cocaine priming precipitated cocaine seeking outside the home but not at home (Fig. 7).
5. It is important to notice that rats were able to update the decision utility of a given lever when one drug was substituted for another. When rats that had worked more vigorously for heroin at home than outside the home were shifted to amphetamine self-administration (after a period of washout), the opposite pattern was observed, as they took more amphetamine outside the home than at home (Caprioli et al. 2008).

In summary, all procedures indicated that the decision utility of heroin is much greater at home than outside the home, whereas the decision utility of cocaine seeking is much greater outside the home that at home. That is, the context exerted *opposite* modulatory influence on the decision utility of heroin versus cocaine.

### Decision utility in humans

The participants in these experiments were female and male clients of Villa Maraini with a long history of both heroin and cocaine misuse (Caprioli et al. 2009; Badiani and Spagnolo 2013). The participants were asked to report on the preferred setting of heroin versus cocaine use. The majority of participants reported to use heroin mainly at home but cocaine mainly outside the home. That is, setting preferences for heroin were *opposite* to those for cocaine. It should be noticed that setting preferences were not the result of practical constraints, as identical patterns were observed in individuals using the same route of administration (intravenous injection, insufflation, or inhalation) for both drugs (Badiani and Spagnolo 2013). It is also important to point out that in most cases cocaine and heroin co-abusers prefer to use the two drugs independently (e.g., Leri et al. 1994; Badiani and Spagnolo 2013). Badiani and Spagnolo (2013) found that only 11% of a population of drug users (also recruited among the clients of Villa Maraini) used ‘speedball’ (a slang term indicating the use of the two drugs at the same time or in close temporal sequence) at one time or another.

## Why does the context influence in opposite directions the experienced utility and decision utility of heroin versus cocaine?

The findings summarized above were initially a source of surprise to us, as they were at odds with the results of previous work concerned with drug-induced psychomotor sensitization, which is thought to reflect the sensitization of mesolimbic dopaminergic transmission (Robinson and Berridge 1993). Indeed, these earlier studies had shown that the magnitude of psychomotor sensitization to *both* psychostimulants (cocaine and amphetamine) and opioids (morphine and heroin) is increased when these drugs are administered outside the home relative to when they are administered at home (Badiani et al. 1995a, b, c, 1997, 2000; Browman et al. 1998a, b; Crombag et al. 1996, 2000; Fraioli et al. 1999; Ostrander et al. 2003; Paolone et al. 2003, 2007). The fact that the modulatory influence of environment on psychomotor sensitization did not vary as a function of drug class was fully consistent with theoretical models centered on the notion that dopaminergic transmission represents the shared substrate of action for addictive drugs (e.g., Wise and Bozarth 1987; Robinson and Berridge 1993; Nestler 2004; Covey et al. 2014; Volkow et al. 2017). The ability of context to influence in *opposite* directions both experienced and decision utility of heroin versus cocaine not only indicates a fundamental dissociation between drug-induced psychomotor sensitization and drug utility, but also calls for a theoretical model that dispenses with unitary notions of drug reward.

We have proposed that any such model must consider: (1) the complexity of central and peripheral actions of drugs, independent of their euphorigenic effects; and (2) the fact that drug effects do not develop in a psychophysiological vacuum. In particular, we have proposed an emotional appraisal model of drug reward, according to which the setting of drug use provides an ‘ecological backdrop’ against which the central and peripheral effects of drugs are appraised (Badiani 2013). This idea builds on Bindra’s emphasis on *stimulus configurations* as the critical motivational drivers of behavior (e.g., “Movements are instigated not by target stimulus features alone but by target stimulus features embedded in proper background stimulus contexts”; Bindra 1976, pp. 59–60). We have extended the notion of background to include drug effects different from the rewarding effects (the target stimulus features).

The central idea of our model is that under certain conditions, there is *mismatch* between some of the effects of the drug and background information. Cocaine, for example, produces a state of arousal by activating central noradrenergic transmission and increases heart and respiratory rate by activating the sympathetic nervous system (Billman 1995; Sofuoglu and Sewell 2009; Maceira et al. 2014). In contrast, heroin depresses the central nervous system and produces parasympathomimetic effects, including the bradycardia (Haddad and Lasala 1987; Thornhill et al. 1989; Nilsson et al. 2016). Although these ‘noneuphoriant’ effects of drugs are usually neglected by theoretical frameworks of drug reward, it is obvious that they represent a set of interoceptive stimuli that overlap in time not only with ‘drug euphoria’ but also with incoming environmental stimuli. This gives rise to the possibility of a *mismatch* between interoceptive and exteroceptive information. When cocaine is taken at home, for example, interoceptive information signals a state of arousal, whereas exteroceptive information signals a quiet, safe environment, hence a mismatch (henceforth, this term will be used exclusively to indicate interoceptive/exteroceptive mismatch). A mismatch might also occur when heroin is taken outside the home, given that the interoceptive information signaling a state of sedation and relaxation conflicts with exteroceptive information signaling exciting, potentially dangerous contexts. We have proposed that in the presence of such mismatches, the experienced utility (both instant and remembered utility) of drugs would be reduced, relative to conditions in which there was no such a mismatch (Badiani 2013). The change in remembered utility would be reflected in a parallel change in decision utility.

Interestingly, in the study by Badiani and Spagnolo (2013), the rationale provided by the small subset of participants using heroin in combination with (or more often after) cocaine was to minimize the anxiogenic effect of cocaine. Consistent with our hypothesis, this occurred more frequently at home (where the anxiogenic effect would be more unpleasant): speedball was used at home in 59.1% of cases and outside the home in 31.8% of cases (in 9.1% of cases, there was no preference for one setting or the other).

The results of studies with other classes of drugs having sedative effects, such as alcohol, or activating effects, such as ketamine, are consistent with our hypothesis. Most heavy drinkers (Nyaronga et al. 2009) and rats (Testa et al. 2011) prefer in fact drinking at home rather than outside the home. In contrast, both humans (De Luca et al. 2012) and rats (De Luca and Badiani 2011) prefer using ketamine (which has sympathomimetic effects; Bevan et al. 1997) outside the home rather than at home.

In the next section, we will discuss in what way a *mismatch* might affect the rewarding effects of heroin and cocaine.

## Implications for computational neuroscience

Can available computational models of *drug reward*^5^ account for the findings reviewed here? A necessary premise for any answer to this question is that computational models of *drug reward* must be kept distinct from computational models of *drug addiction* (e.g., Redish et al. 2008; Keiflin and Janak 2015), which deal with the loss of control over drug use. It is also important to distinguish computational models of *drug reward* from computational models of *reward-related learning*. As noted by Berridge et al. (2008), “Current computational models predict reward based solely on learning. Real motivation involves that but also more.” Given the definition of reward provided in the early sections of this paper, we are interested in models that include “explicit representation or cognitive model of the UCS and their place in the world” (Berridge 2012, p. 1127).

Clearly, all models of drug reward based on the notion that the experienced utility and/or the decision utility of all addictive drugs depends on unitary mechanisms of action cannot explain the findings reviewed here. For example, models that posit that the rewarding effects all substances of abuse depend entirely on the facilitation of dopamine transmission (e.g., Keiflin and Janak 2015) can hardly explain why heroin and cocaine should be appraised so differently by rats and humans.

Finally, to account for our findings, a model must include a component that could mediate *the substance-specific influence of setting on drug reward*. To the best of our knowledge, there are only two models that, with some tweaking, might accommodate for this last requirement.

**Fig. 8:**
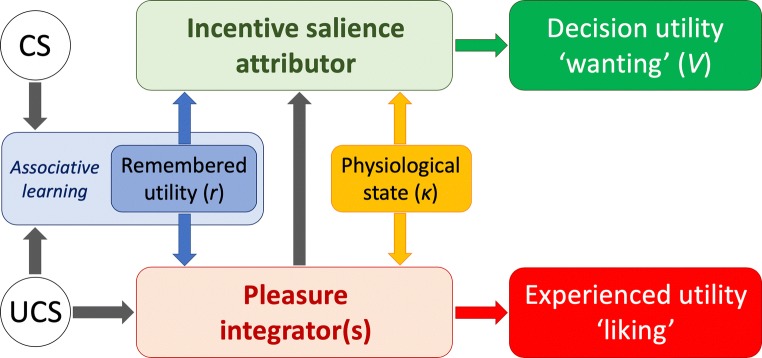
Schematic representation of the Michigan model (Robinson and Berridge 1993; Berridge 2012). Not all features of the model are reproduced here (e.g., reboosting). The computational version of the incentive salience component of the model (Zhang et al. 2009) calculates the decision utility of a stimulus S at time t with the formula: Ṽ(St) = rt x κ + γV(St+1), where γ is a temporal discount factor. See text for details

The first of these models is that originally proposed by Robinson and Berridge (1993). A formal computational version of (the incentive salience component of) this model was later proposed by Zhang, Berridge, and colleagues (Zhang et al. 2009; Dayan and Berridge 2014). As the researchers who proposed this model were based at the University of Michigan, this model will be referred to as the Michigan model (see Fig. 8). More recently, Keramati and colleagues (Keramati and Gutkin 2014) proposed a computational model of homeostatically regulated reinforcement learning (HRRL), which, according to the authors represents a “normative generalization” of the Zhang’s equation (Keramati et al. 2017, p. 149). However, the HRRL model is based on the notion of drive reduction that the Michigan model emphatically rejects (Berridge 2004). While the Michigan model clearly predicts that the ‘value’ of a UCS depends on its hedonic effect (➔ remembered utility), the Keramati/Gutkin’s model posits that it reduces a pre-existing homeostatic ‘need.’ In the case of the early encounters with a drug, it is not clear what exactly that need might be. Thus, we will not consider this model further.

The main feature of the Michigan model is that it includes separate processing systems for incentive salience attributor ➔ ‘wanting’ (i.e., ‘pure’ decision utility^6^) and for a pleasure integrator ➔ ‘liking’ (i.e., experienced utility). Zhang, Berridge, and colleagues (Zhang et al. 2009) proposed a computational version of the incentive salience attributor. Zhang’s equation includes *r*, a learned cache value of the experienced utility (that is, remembered utility) of unconditioned, or unconditional, stimuli (UCSs),^7^ which becomes associated with the stimulus features of the UCS and of conditioned, or conditional, stimuli (CSs), through a process of incentive learning. On subsequent encounters, UCS/CS will directly activate the incentive salience attributor eliciting ‘wanting.’ It must be stressed that although the authors of the Michigan model have placed particular emphasis on postlearning components of Pavlovian motivation, from a formal point of view, the model does not distinguish between CSs and UCSs if not for the ability of the latter to directly access the pleasure integrator.

The gain of the incentive salience attributor (as well as of the ‘pleasure integrator’) can be raised or decreased by dynamic fluctuations in neurobiological states that are relevant to the motivational value of a given UCS/CS (e.g., physiological drives, such as hyper-/dehydration or hunger/satiety, or pharmacological manipulations that impinge directly onto the machinery of the two processing systems). These modulatory influences, which do not require new learning, are represented by the gating parameter *κ*, which serves a purely computational function, as it mediates incoming information of the most disparate nature. The final decision utility of the UCS/CS is represented by *Ṽ.* The decision utility of a stimulus *S* at time *t* is: *Ṽ*(*S*_*t*_) *= r*_*t*_ × *κ* + *γV*(*S*_*t* + 1_), where *γ* is a temporal discount factor. Hence, in the case of heroin or heroin cues (*H*) at time *t*, *Ṽ*(*H*_*t*_) *= r*_*t*_ × *κ* + *γV*(*H*_*t* + 1_).

The most ‘robust’ component of the Michigan model is the incentive salience attributor. Its core neurobiological substrate is represented by the mesolimbic dopaminergic system and related circuitry. This system, besides encoding the learned motivational salience of UCS/CS, represents a direct target for drugs that modulate dopaminergic transmission (Berridge 2012). Indeed, it has been proposed that all addictive drugs can directly elicit wanting, by activating dopaminergic transmission, in the absence of pleasure, that is, with little or no experienced utility (Robinson and Berridge 1993). Furthermore, by repeatedly activating dopaminergic transmission, addictive drugs can sensitize this system and amplify the response of the incentive salience attributor to drugs and drug-paired cues.

In contrast, the exact nature of the ‘pleasure integrator’ of the Michigan model is more nebulous. Work done by Berridge and colleagues suggests that orosensory pleasure elicited by sweet tastes is processed by a distributed system including a number of interconnected ‘hedonic hotpots’ (for a review, see Berridge 2012). To the best of our knowledge, there is no evidence that the same network of ‘hedonic hotspots’ is also responsible for the computation of the hedonic effects of drugs (and in particular of psychostimulant drugs) in rats. It is fair to say the existence of a ‘common neurobiological currency’ of experienced utility is mostly predicated on correlational data from heterogeneous neuroimaging studies in humans (for a review, see Berridge and Kringelbach 2013), the interpretation of which is to say the least problematic, owing not only to the intrinsic limitations of imaging techniques but also to the reliance on the response to cues instead than on actual hedonic experiences.

Other features of the ‘pleasure integrator’ are less than clear. It is not clear, for example, what type of drug effects is processed by the integrator to compute overall experienced utility. Does the ‘pleasure integrator’ compute only the instant utility of rapid surges of drug concentration in the brain, such as the intense, orgasmic euphoria produced by intravenous injections of heroin or cocaine or the ‘buzz’ produced by a puff of tobacco smoking? Or it does it also compute the experienced utility of the TEO of drug use, which depends on a number of neurobiological processes independent of euphoria? For instance, are the feeling of contentment (well-being) and the sedative, benumbing, anxiolytic, and analgesic effects of heroin (Jaffe et al. 1997) processed by the same ‘pleasure integrator’ responsible for the immediate euphorigenic effect? Is it possible that distinct integrators process discrete types of heroin utility (e.g., euphoria, contentment, sedation, benumbment, anxiolysis, analgesia), which are then compounded into a common neurobiological currency of remembered experienced utility? And finally, how many ‘pleasure integrators’ are there? Is there a single ‘pleasure integrator’ for all addictive drugs or are there many? Notice that although these issues remain to be addressed, they do not challenge the fundamental structure of the Michigan model. Thus, to simplify the discussion in the following paragraphs, we will provisionally assume that a single integrator computes *all drug effects relevant to the hedonic experience*. It is beyond the scope of this paper to consider alternative possibilities.

Finally, from a conceptual point of view, the Michigan model can easily accommodate the existence of distinct ‘*κ* spaces’ qualitatively unique to the appropriate UCS, each able to modulate in a specific manner only the response to its own UCS and related CSs. Thus, it would be reasonable to assume the existence of distinct *κ* spaces for heroin and cocaine. The Zhang’s equation, however, does not offer a formal computational version of these multiple *κ* spaces, and the authors acknowledge this limitation of the model (Zhang et al. 2009, p. 12).

Does the Michigan model agree with the results of the rat’s studies reviewed in the previous sections and with our working hypothesis? The following analysis makes two working assumptions. First, we will assume that in a virtual landscape without *κ* space(s), the cache values for the doses of heroin (25 μg/kg) and cocaine (400 μg/kg) used in our comparative experiments (Caprioli et al. 2009; Celentano et al. 2009; Avvisati et al. 2016; De Luca et al. 2019) are the same. This assumption seems reasonable not only because these doses were selected on the basis of dose–effect curves (Caprioli et al. 2007a, 2008) but because, as shown in Fig. 5, when the groups at home and outside the home are collapsed, approximately the same proportion of rats chooses heroin and cocaine (36 and 43%, respectively). The second working assumption is that there are distinct *κ* spaces for heroin and cocaine.

We will first consider experienced utility and then decision utility.

### Parameter *κ* and experienced utility

Although the Zhang’s equation is concerned exclusively with the attribution of incentive salience, it is clear that the Michigan model can easily predict greater utility of heroin at home than outside the home and vice versa for cocaine (see Figure 2 in Robinson and Berridge 1993 and Figure 1 in Berridge 2012). It is sufficient to postulate that interoceptive and exteroceptive information impinge onto the integrator via the parameter *κ*. The *κ* value for heroin (*κ*_H_), for example, would take different values depending on whether a mismatch is absent (*κ*_H,no-mismatch_ = 1) or present (*κ*_H,mismatch_ < 1). It follows that the ‘pleasure integrator’ will yield greater liking for heroin at home then outside the home, and greater liking for cocaine outside the home then at home. These differences in experienced utility will translate into parallel differences in the remembered utility (*r*) of heroin versus cocaine cues. We have seen in a previous section that the activity of a fronto-striato-cerebellar network mirrors the changes in the remembered utility of heroin versus cocaine, as a function of setting, in drug users (De Pirro et al. 2018). It is too early to speculate whether this network might serve as a ‘pleasure integrator’ or simply encode the parameter *κ*.

### Parameter *κ* and decision utility

As stated above, the differences in the experienced utility of heroin and cocaine as a function of setting will translate in differences in the cache value *r* of the respective UCS/CS leading to difference in *V*. These differences will be further magnified at the level of decision utility owing again to parameter *κ*. Indeed, the incentive salience attributor will yield *V* = 1 when *κ*_no-mismatch_ = 1, and *V* < 1 when *κ*_mismatch_ < 1. This would explain why the rats self-administered and worked harder for heroin at home than outside the home and vice versa for cocaine during the acquisition of drug self-administration (Caprioli et al. 2007a; De Luca et al. 2019; see Fig. 4).

In contrast, in the choice sessions illustrated in Figs. 3D, 5, the rats were confronted with a completely new situation relative to the acquisition phase. During the acquisition phase (Fig. 3A–C), the rats received the same drug (heroin or cocaine) by pressing on alternate sessions on the left or the right lever (De Luca et al. 2019). Clearly, by the end of training, the decision utility of the two levers was identical. During the choice session, both levers were made available simultaneously, but one was paired with heroin and the other with cocaine. Depending on the setting, the rats tended to prefer heroin to cocaine (at home) or cocaine to heroin (outside the home), indicating a shift in the decision utility of the two levers.

One obvious possibility is that the change in experienced utility leads to a re-evaluation of the CS (lever+cues) and a new *r* value, which implies new learning. Alternatively, can the Zhang’s equation account for the change in decision utility for the two levers, independent of new learning? Apparently yes, if, as already discussed, there are no intrinsic qualitative or quantitative differences in the experienced utility of a single dose of heroin or cocaine.^8^ For example, during training with cocaine outside the home, *κ*_C,no-mismatch_ = 1 and *V* = 1 for both levers. When, during the choice phase the rat (purely by chance) completes the task on the lever that now triggers an infusion of heroin, the equation will yield *V* < 1 because of *κ*_H,mismatch_ < 1, whereas the decision utility of the cocaine-paired lever remains the same (*V* = 1). Thus, rats outside the home will tend to choose cocaine over heroin. The opposite will occur in rats trained with cocaine at home. In this case, during training, *κ*_mismatch_ < 1 and *V* < 1 for both levers, but during the choice phase, the decision utility of the lever paired with heroin will increase (*κ*_H,no-mismatch_ = 1 and *V* = 1). Thus, rats at home will tend to prefer heroin over cocaine. In summary, in the words of Zhang and colleagues (Zhang et al. 2009, p. 5), it appears that “specific *κ*’s determine what to ‘want’.”

Finally, we will consider the ability of Zhang’s equation to account for the opposite modulatory influence of setting on the ‘priming’ effect of heroin versus cocaine in rat model of relapse, as previously described (see Fig. 7). In this experiment non-contingent doses of heroin or cocaine were administered to rats after an extinction phase, during which they were repeatedly given access to the CS (cue lights, lever extension, vehicle infusion) but not to the UCS (the drug). Berridge and colleagues (Robinson and Berridge 1993; Berridge 2012) have proposed that operant conditioned behavior requires ‘reboosting’ of ‘wanting,’ which serves as “an incremental mechanism of incentive salience maintenance” occurring at each CS–UCS rewarded trial. In the absence of the UCS, ‘deboosting’ of incentive salience occurs, leading to a progressive decrement in responding (but never to its complete disappearance). However, responding might resume under extinction conditions—that is, during unrewarded trials—if a small ‘priming’ dose of the drug is administered at the start of the session (Robinson and Berridge 1993; Berridge 2012). The priming effects of drug are included in the parameter *κ*, thus representing a special case of what has been discussed in the previous paragraphs: *κ*_no-mismatch_ = 1 and *V* = 1 for heroin priming at home and cocaine priming outside the home versus *κ*_mismatch_ < 1 and *V* < 1 for heroin priming outside the home and cocaine priming at home.

## Dopamine and incentive salience attribution

As discussed in the previous sections, from a purely conceptual point of view, the Michigan model seems robust enough to handle the substance specificity of environmental influences on both experienced utility and decision utility. However, some difficulties arise when the neurobiological nature of the Michigan model is considered. We will focus here first on parameter *κ* and then on the role of the mesolimbic dopaminergic system as the core mechanism of incentive salience attribution.

### Parameter *κ* and dopamine

Although Zhang, Berridge, and colleagues (Zhang et al. 2009; Berridge 2012; Berridge and O’Doherty 2014) have made it sufficiently clear that the parameter *κ* is a purely computational device, it remains to be explained what type of neurobiological mechanisms are responsible for gating the activity of the incentive salience attributor. It is possible to distinguish proximal and distal components of parameter *κ*. The Michigan model clearly posits that the proximal mechanism is represented by fluctuations of dopamine levels. The distal mechanisms must necessarily change depending on the nature of the relevant brain state. Berridge (2012), for example, has discussed in detail the neurobiological mechanisms responsible for the modulatory influences of natural appetites and satiety on the response to orosensory stimuli. Here, we are concerned only with the mechanisms implicated in the effect of heroin versus cocaine primings and of interoceptive/exteroceptive mismatches.

In the case of drug priming, we are dealing with the simplest version of parameter *κ*, as in this case there are no distal mechanisms: drugs simply plug into the incentive salience attributor by activating dopaminergic transmission. Indeed, the Michigan model attributes the ability of drug primings to reboost the incentive salience attributor to the activation of dopaminergic transmission (Robinson and Berridge 1993; Berridge 2012). It is commonly assumed that all substances of abuse increase dopaminergic transmission (e.g., Di Chiara and Imperato 1988; Covey et al. 2014), albeit via different mechanisms of action. Cocaine induces dopamine overflow in the terminal regions of mesotelencephalic dopaminergic system by blocking the dopamine-reuptake transporter (for reviews, see Johanson and Fischman 1989; Kuczenski et al. 1982). Heroin is thought to increase dopaminergic concentrations in the same regions indirectly by binding mu-opioid receptors (MOR) in the ventral tegmental area and substantia nigra, hence disinhibiting dopamine-releasing neurons (Gysling and Wang 1983; Matthews and German 1984; Johnson and North 1992), and by increasing the amplitude of phasic relative to tonic dopamine signals (Britt and McGehee 2008). Microdialysis experiments have consistently shown that noncontingent administration of cocaine or, to a lesser extent, of heroin increases extracellular dopamine concentrations over a time scale of several minutes (e.g., Hemby et al. 1995; Marinelli et al. 1998; Pattison et al. 2011, 2012; Gottås et al. 2014). Less clear-cut are the microdialysis data concerning the changes in dopamine concentrations during self-administration. In the case of heroin, for example, some studies reported modest increases in dopamine (e.g., Wise et al. 1995) and others did not (Hemby et al. 1995).

Most relevant to understand the direct effects of self-administered drugs on dopamine transmission are the studies using voltammetric methodology, which monitor dopaminergic activity on a second or subsecond scale. These studies have shown that in rats self-administering cocaine or heroin, the dopamine signal decreases sharply immediately after the delivery of either drug, but not after a sham vehicle infusion (Gratton and Wise 1994; Kiyatkin 1995; Gratton 1996; Stuber et al. 2005; Cameron et al. 2014). A similar decrease is produced by single passive infusions of similar doses of heroin or cocaine, like in the case of the priming injections used in our experiments (see Fig. 5). Electrophysiology studies support the findings from voltammetry experiments. Kiyatkin and Rebec (1997), for example, monitored the activity of presumed dopamine neurons in the ventral tegmental area during heroin self-administration and found “a transient inhibition of DA activity correlated with heroin reward” followed by DA activation during “heroin-seeking behavior.” The finding of the studies cited above are not seriously challenged by the claims of increased dopamine activity produced by cocaine self-administration (e.g., Phillips et al. 2003; Aragona et al. 2009; Willuhn et al. 2011). In these studies, the infusion (over a period 3–6 s) is paired with exposure to conditioned stimuli with much longer duration (20 s). This makes it impossible to distinguish the relative contribution of UCS versus CS (see Rescorla 1967). Furthermore, the increase in dopamine begins almost immediately after exposure to the UCS/CS and lasts only a few seconds, which is not compatible with the pharmacodynamics of drug actions in the CNS, whereas it is perfectly in sync with the physiology of cue reactivity. In summary, the immediate effect of addictive drugs on dopaminergic activity does not appear to be consistent with the notion that the modulatory influence of parameter *κ* is necessarily mediated by an increase in dopaminergic transmission.

The possible neurobiological mechanisms responsible for the blunting influence of mismatch on decision utility of heroin and cocaine are much more complex and have been the object of a detailed discussion in an earlier paper (Badiani 2013). Briefly, we proposed that the basolateral amygdala (BLA), which plays a central role in the emotional appraisal of interoceptive and exteroceptive stimuli (including central and peripheral drug effects, such as changes in heart rate and respiratory rate), is crucially positioned to detect eventual affective mismatches (Sah et al. 2003; McGaugh 2004; Salzman and Fusi 2010; Murray 2007; Tamietto and de Gelder 2010; Critchley and Harrison 2013). The BLA can then transfer this information to the brain regions that directly control goal-directed behavior, such as the striatal complex and prefrontal cortex (Murray 2007). Studies using in situ hybridization of c-fos mRNA as an index of neuronal activation in the rat brain lend some support to this hypothesis (Day et al. 2001). Cocaine and amphetamine produce in fact much greater activation of the BLA when administered outside the home (no mismatch) than when administered at home (mismatch). A similar pattern was observed in the dorsal striatum and in the nucleus accumbens (Badiani et al. 1998; Uslaner et al. 2001a; Ostrander et al. 2003; Hope et al. 1992). In contrast, the setting appears to modulate in a very different, sometimes *opposite*, manner the effects of opioids, such as morphine and heroin (Ferguson et al. 2004; Paolone et al. 2007; Celentano et al. 2009). Particularly relevant here is finding that, within the striatum, cocaine and amphetamine increases the activity of the D2+/enkephalin+ medium spiny neurons (MSNs) to a much greater extent when administered outside the home than when administered at home (Badiani et al. 1999; Uslaner et al. 2001b), whereas the opposite occurs with morphine (Ferguson et al. 2004). In turn D2+/enkephalin+ MSNs indirectly disinhibit the subthalamic nucleus, which has been implicated in reward and decision-making (Zénon et al. 2016; Pelloux et al. 2018). These data provide a tentative set of distal mechanism responsible for the opposite influence of setting on the decision utility of opioids versus psychostimulant drugs. Even in this case, there is some reason to doubt that the proximal mechanism is represented by dopaminergic transmission. Microdialysis experiments, conducted in parallel with the in situ hybridization experiments reviewed above, have shown virtually no difference in amphetamine-induced dopamine overflow in the nucleus accumbens core and shell and other subregions of the striatal complex of rats tested at home versus rats tested outside the home (Badiani et al. 1998, 2000). Taken together, these findings seem to suggest that at least some of the inputs included in parameter *κ* might modulate decision utility downstream from dopamine transmission.

### Decision utility ‘wanting’ and dopamine

The voltammetry studies cited in the previous section have shown that the dopamine signal progressively increases at the presentation of the CS marking the onset of each trial of cocaine (Gratton and Wise 1994; Gratton 1996) or heroin (Kiyatkin 1995) availability. This and other evidence support the notion that dopamine encodes either a prediction error signal or the incentive salience properties of drug-paired cues (e.g., Schultz et al. 1993; Montague et al. 1996; Berridge 2007; Flagel et al. 2011; for a broader discussion of the role of dopamine in learning and motivation see Berke 2018). Yet, it is not clear to what extent this contributes to the decision utility of drugs. Comparative studies have shown that the while the reinforcing effects of cocaine are greatly reduced by lesions of the dopaminergic system, by dopamine receptor antagonists, or by RNA interference of dopamine D1 receptors, this is not the case for heroin (Ettenberg et al. 1982; Pettit et al. 1984; Gerrits et al. 1994; Gerrits and Van Ree 1996; Pisanu et al. 2015). Thus, even though, as mentioned previously, noncontingent administrations of heroin can produce negligible to modest increases in dopamine concentrations in the nucleus accumbens, as measured via microdialysis, it is unlikely that this contributes in a significant manner to its decision utility. In this respect, it is remarkable that the reinforcing effects of opioid receptor agonists do not correlate at all with their ability to increase dopaminergic concentrations, as measured by microdialysis. The reinforcing effect of 6-acetylmorphine (an active metabolite of heroin), for example, is comparable to that of heroin (Avvisati et al. 2018) even though the former induces much greater and faster dopamine increases than the latter (Gottås et al. 2014). Even more striking is the fact that the selective kappa-opioid agonist RU 51599 has greater reinforcing effect than heroin (at low but not at high workload) even though it greatly decreases dopamine concentrations in the nucleus accumbens (Marinelli et al. 1998).

## Conclusions

We have reviewed here a series of translational studies demonstrating that the experienced utility and decision utility of heroin and cocaine are modulated in opposite direction by the circumstances of drug use. This confirms the importance of settings in modulating drug reward (see Zinberg 1984; Crombag and Shaham 2002; Caprioli et al. 2007b; Ahmed et al. 2018) and challenges prevailing unitary theories of drug reward and drug addiction (Badiani et al. 2011; Badiani et al. 2018; Badiani 2013). Of course, the differences between the rewarding effects of heroin and cocaine (not to mention other addictive drugs) go well beyond those observed under the influence of environmental context (for reviews, see Caprioli et al. 2007b; Redish et al. 2008; Ikemoto 2010; Badiani et al. 2011, 2018; Peters et al. 2013; Nutt et al. 2015).

Most important, our findings and much of the evidence concerning the role of dopamine in heroin reward (Ettenberg et al. 1982; Pettit et al. 1984; Gerrits et al. 1994; Gerrits and Van Ree 1996; Pisanu et al. 2015) appear to challenge the notion that mesolimbic dopamine transmission encodes the final decision utility of all addictive drugs, which underpins most model of drug reward (e.g., Wise 1980; Wise and Bozarth 1987; Nestler 2001, 2004; Sulzer 2011; Covey et al. 2014; Keiflin and Janak 2015; Volkow and Morales 1995). Thus, our findings have implications for the development of robust, context-sensitive, computational models of *drug reward*.

It appears of the extant models that none is fully compatible with the results discussed above. However, we have shown that with some tweaking the architecture of the Michigan model (Robinson and Berridge 1993; Berridge 2012) and its computational version (Zhang et al. 2009; Dayan and Berridge 2014) can accommodate most of our findings, except for the critical role that this model attributes to dopamine. In this respect, however, it is worth noticing that in the graphic representations of the Michigan model, no specific neurobiological label is attached to its components, including the incentive salience attributor (e.g., Figure 2 in Robinson and Berridge 1993, and Figure 1 in Berridge 2012; but see Figure 1 in Berridge and Robinson 2016). More explicitly, in the original formulation of their model, Robinson and Berridge (1993) stated (note 4, p. 275): “Regardless, we want to emphasize that the Incentive-Sensitization Theory of Addiction *does not require that the sole or even primary site of drug-induced neuroadaptations responsible for craving specifically be on dopamine neurons*. If it is not, then our assignment of sensitization of incentive salience to dopamine would be incorrect. Nevertheless, the concept that drug craving develops because of sensitization of incentive salience could still be fundamentally correct, but it would be mediated by another, as yet unidentified neural substrate.” It is fair to say, however, that in their papers, Robinson and Berridge place such an emphasis on the central role of the dopaminergic system (e.g., “Berridge’s Incentive Salience Theory: Dopamine as Pure Decision Utility” in Berridge and O’Doherty 2014, p. 341) that it becomes difficult to separate dopamine from the incentive salience attributor. Similar considerations can be made for the role of dopamine in mediating the influence of parameter *κ* on the incentive salience attributor (see Zhang et al. 2009; Berridge 2012).

Whether the Michigan model can be further developed to overcome these apparent limitations (as well as the limitations already acknowledged by the authors; see Zhang et al. 2009; Berridge 2012) is a matter for future research. In any case, we hope that this paper will serve as stimulus to design computational models more attuned to the complex mechanisms responsible for the rewarding effects of drugs in real-world contexts.

## Footnotes

1 Many authors shift more or less surreptitiously from one meaning to another, sometimes within the same paper.

2 It is beyond the scope of this review to provide a critical analysis of these theories.

3 Of course, in principle, there is no conceptual reason to limit the notion of remembered utility (both in the sense of stored information and of retrospective report) to TEOs, as it might equally apply to outcomes with minimal temporal extension (that is, to instant utility).

4 As for other putative indices of *affective* states in rats (e.g., hedonic facial expression in rats, thought to express the orosensory pleasure elicited by sweet tastes; for a review, see Berridge 2003), it is not possible to exclude that USVs reflect instead positive *motivational* states.

5 Notice that models of reward are relevant to drug reward only to the extent that they can account for the specific mechanisms of action of drugs.

6 Which is a prepotent psychological process much more relevant to the compulsive nature of drug taking than higher order cognitive forms of decision-making (wanting in the ordinary sense of the word, according to Berridge and O’Doherty 2014, p. 341).

7 Notice that Ivan Pavlov coined the term “conditioned reflexes to distinguish them from the inborn or unconditioned reflexes,” which are “already completed at birth” (Pavolov 1927, p. 25). That is, an UCS should be able to trigger a response before previous learning, as opposed to CSs. However, as pointed out by Hilgard and Marquis (1961), very soon the term UCS came to be used operationally to indicate “a stimulus that, *at the outset of an experiment* [emphasis by the authors], evokes a regular and measurable response”. Hilgard and Marquis, went on “mentioning specifically that the unconditioned response is not necessarily an unlearned or reflex response to the UCS. In many cases, such as salivation to food placed in the mouth, it is; but in other cases, such as salivation at sight of smell and food in the distance, which Pavlov also used as UCS, it is not.” Thus, a distinction must be made between Pavlov’s original definition of UCSs (which have ‘inborn’ ability to evoke a response) and the operational definition of UCS in the context of conditioning procedures (including Pavlov’s). Theorists interested in explaining real-world phenomena are concerned with the notion of UCS and CS encapsulated by Pavlov’s original definition. Thus, in the context of the Michigan model, a true UCS must have hardwired intrinsic ability to impinge on the pleasure integrator and/or on the incentive salience attributor. In considering the latter possibility, Berridge noted that “it is possible to imagine inserting a UCS value such as, say, *U*_*t*_ value (*U*_*t*_ for unconditioned UCS attractive features) as level of innate attractiveness in place of the learned cache *r*_*t*_ value” (Berridge 2012, p. 1271).

8 If the experienced utility of heroin and cocaine differ qualitatively from each other, it would be difficult to assume that this new information will not lead, again via incentive learning, to a re-evaluation of the learned utility of the relevant CS.
